# Two Tau binding sites on tubulin revealed by thiol-disulfide exchanges

**DOI:** 10.1038/s41598-018-32096-9

**Published:** 2018-09-14

**Authors:** Marlène Martinho, Diane Allegro, Isabelle Huvent, Charlotte Chabaud, Emilien Etienne, Hervé Kovacic, Bruno Guigliarelli, Vincent Peyrot, Isabelle Landrieu, Valérie Belle, Pascale Barbier

**Affiliations:** 1 Aix-Marseille Univ, CNRS, UMR 7281 BIP, Bioénergétique et Ingénierie des Protéines, Marseille, France; 2 Aix-Marseille Univ, CNRS, UMR 7051, INP, Institut de Neurophysiopathologie, Marseille, France; 3 Lille Univ, CNRS, UMR 8576, UGSF, 59000 Lille, France

## Abstract

Tau is a Microtubule-associated protein that induces and stabilizes the formation of the Microtubule cytoskeleton and plays an important role in neurodegenerative diseases. The Microtubules binding region of Tau has been determined for a long time but where and how Tau binds to its partner still remain a topic of debate. We used Site Directed Spin Labeling combined with EPR spectroscopy to monitor Tau upon binding to either Taxol-stabilized MTs or to αβ-tubulin when Tau is directly used as an inducer of MTs formation. Using maleimide-functionalized labels grafted on the two natural cysteine residues of Tau, we found in both cases that Tau remains highly flexible in these regions confirming the fuzziness of Tau:MTs complexes. More interestingly, using labels linked by a disulfide bridge, we evidenced for the first time thiol disulfide exchanges between αβ-tubulin or MTs and Tau. Additionally, Tau fragments having the two natural cysteines or variants containing only one of them were used to determine the role of each cysteine individually. The difference observed in the label release kinetics between preformed MTs or Tau-induced MTs, associated to a comparison of structural data, led us to propose two putative binding sites of Tau on αβ-tubulin.

## Introduction

**Figure 1:**
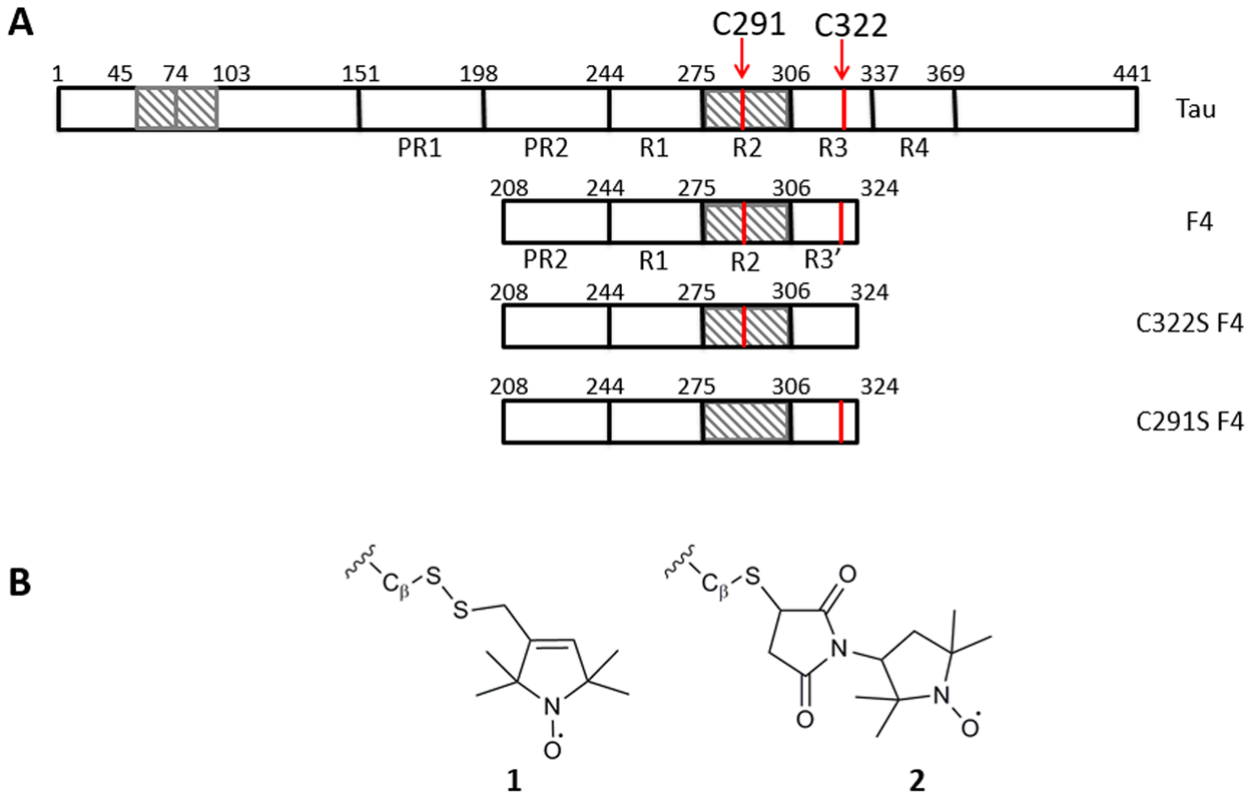
Schematic representation of Tau and F4 fragments sequences and of spin labels. (**A**) Hatching boxes represent exons 1 and 2 in the N-terminal domain and exon 10 in the MTB domain differencing the Tau isoforms by alternative splicing of its mRNA. PR1 and PR2 boxes are proline riche regions. R1, R2, R3, R4 are the imperfect MT-binding repeats. Arrows indicate the 291 and 322 positions of the natural cysteines. (**B**) Chemical structures of grafted MTSL (**1**) and proxyl (**2**) spin labels.

In eukaryotic cell, the microtubule (MT) cytoskeleton constitutes a functional network involved in a diverse range of cellular functions such as mitosis and meiosis, motility, morphogenesis and intracellular trafficking of macromolecules and organelles. The core component of MT is constituted of 13 protofilaments of a head-to-tail assembly of a heterodimer of 50 kDa α- and β-tubulin proteins, self-assembling themselves to form a cylinder of 25 nm diameter and some micrometer long. MTs are highly dynamic, and undergo rapid stochastic transitions between growth and shortening phases, due to the association and/or dissociation of tubulin dimers from the MTs ends^1^. This process is regulated by a family of proteins called Microtubules Associated Protein such as Tau. Tau induces and stabilizes the formation of the Microtubule cytoskeleton^2^ and plays an important role in neurodegenerative diseases such as Alzheimer disease^3–5^. In nervous central system, Tau is constituted of six isoforms encoded by a single gene^6^. Each isoform (from 352 and 441 amino acid residues) presents either three (Tau-3R) or four (Tau-4R) imperfect MT-binding (MTB) repeats located in the C-terminal half of the protein, and zero to two inserts located in the N-terminal portion^7,8^. The MTB repeats are flanked upstream by a basic proline-rich regulatory region, which can enhance the binding (Fig. 1A)^9–12^. Using NMR, we demonstrated that the amino acids involved in the binding of Tau on MTs stabilized by Taxol, a MTs stabilizing anticancer agent, are located between Ser214 and Glu372^13^. Similar binding region was proposed by Kadavath *et al*., combining NMR and biological approach^14^.

Even if the MTs binding region of Tau has been determined, numerous studies tried to understand where and how Tau binds to MTs and the model of Tau:MTs interaction still remains a topic of debate. Kar *et al*. proposed that the MTB region is located inside the microtubule in the vicinity of the Taxol site localized on β-tubulin in the inner MTs surface^15,16^. These authors suggested that the proline rich domain binds longitudinally along a protofilament and the N-terminal part projects outside the MTs wall^17,18^. This hypothesis was reinforced by the finding of different kinetics of Tau binding on Microtubule in presence or in absence of Taxol suggesting that they share similar binding site^19^. Another study showed that Tau binds exclusively to the outside surface of MTs, not only along protofilaments but also across^20,21^. Others proposed a longitudinal binding of Tau along the protofilament outside the MT wall^22–26^. More recently, Tau was found to bind at the interface between two adjacent tubulin heterodimers, near the binding site of vinca alkaloids, a family of MTs destabilizing’s agents^27^. The difficulty to determine the location and geometry of Tau binding to MTs is probably linked to the fact that Tau is an intrinsically disordered protein (IDP), lacking a precise 3D structure in solution and remaining highly flexible when bound to the MTs^13,20,27,28^. However, different local structural changes of Tau upon binding to tubulin have been reported and the subject remains highly controversial. The formation of α helical segments in the MTB repeat has been reported by combining Fluorescence Correlation Spectroscopy (FCS) and acrylodan fluorescence screening^29^, whereas NMR studies on a Tau fragment (from amino acids 267 to 312) demonstrated that the conserved hexapeptides at the beginning of Tau R2 and R3 repeats adopt a hairpin conformation^14^. In this context, obtaining structural information on Tau binding on MTs and the location of Tau binding site on tubulin is thus highly challenging.

The aim of this study is to probe Tau dynamics upon binding to MTs either with preformed Taxol-stabilized MTs or under more physiologically relevant conditions in which Tau is used as MTs inducer. One powerful technique to study protein dynamics is Site Directed Spin Labeling combined with EPR spectroscopy (SDSL-EPR)^30–32^. Based on the grafting of paramagnetic labels (nitroxide derivatives) usually on cysteine residues, this approach is very sensitive to identify structural transitions particularly in the case of flexible proteins such as IDPs^31^. We took advantage of the fact that the longest isoform of Tau (441 aa) contains two natural cysteines, located in the MTB region (C291 and C322), that we used as targets for spin labeling (Fig. 1A). Using a maleimido-functionalized spin label we confirmed that Tau remains, in the vicinity of the cysteine regions, very flexible in its bound form to MTs. Using a methanethiosulfonate-functionalized nitroxide, an unexpected label release was observed upon binding to tubulin revealing for the first time a thiol-disulfide exchange between Tau and tubulin dimers. To go further, we used Tau fragments (from amino acids 208 to 324) having the two natural cysteines or variants containing only one of them to determine the role of each cysteine individually (Fig. 1A). Analysis of the kinetics of this thiol-disulfide exchange allowed us to evidence two Tau binding sites on tubulin and to point the involvement of specific cysteines in the tubulin dimer.

## Results and Discussion

### Functional aspects of labeled Tau

**Figure 2:**
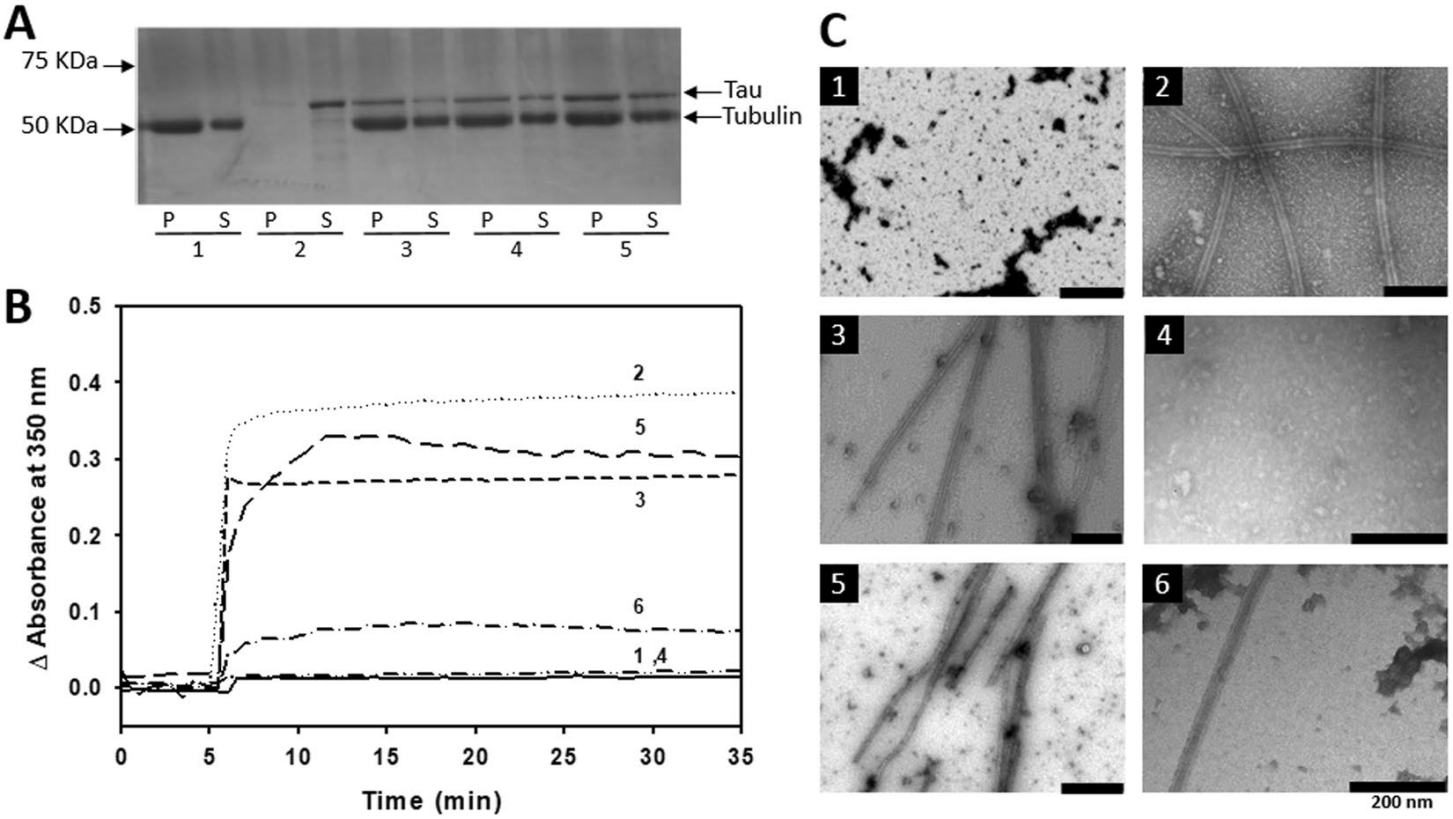
Interaction characterization of labeled Tau with Taxol-stabilized Microtubules. (**A**) SDS-PAGE of Tau^MTSL^ interaction with 5 µM Taxol-stabilized Microtubules. Lane 1 represents tubulin amount (5 µM before centrifugation), respectively in pellet P (MTs) and in supernatant S (free tubulin), in absence of Tau^MTSL^. Lane 2 represents Tau^MTSL^ amount in the pellet P and the supernatant S in absence of tubulin ([Tau] = 5 µM). Lanes 3, 4, 5 represent tubulin and Tau^MTSL^ in the pellet P and in the supernatant S with 2.5, 3.5 and 5 µM of Tau^MTSL^ respectively. (**B**) Turbidimetry time course of MTs formation at 37 °C of (1) 5 µM tubulin alone, (2) in presence of 5 µM of Tau with 1 mM TCEP, (3) in presence of 5 µM of Tau^MTSL^, (4) in presence of 5 µM of Tau^proxyl^, (5) 20 µM tubulin in presence of 5 µM of Tau^proxyl^ and (6) in presence of 5 µM of alkylated Tau. Buffer: NaPi 20 mM pH 6.5 GTP 0.1 mM. Tau proteins have been added after 5 minutes. (**C**) Electron micrographs of the different samples. The scale bar represents 200 nm and the numbers correspond to the samples indicated in (**B**).

Tau was labeled with MTSL on its two natural cysteines localized in the MTB domain, more precisely in the second and third repeat (R2 and R3) at positions 291 and 322 (Fig. 1A,B) (referred to as Tau^MTSL^). To check that Tau binding on Taxol-stabilized MTs is not modified by Tau labeling on these sites, co-sedimentation assay was performed (Fig. 2A). MTs formation was achieved by adding a 1.5 molar excess of Taxol on tubulin dimer. Different concentrations of Tau^MTSL^ were mixed with Taxol-stabilized MTs at 20 °C and centrifuged through a glycerol cushion. The supernatant that represents the amount of free Tau and tubulin and the suspended pellet that represents Tau bound to MTs and tubulin in MTs were analyzed using SDS-PAGE. In absence of Tau^MTSL^, tubulin was found both in the pellet (MTs) and in the supernatant (tubulin dimers) (Fig. 2A, lane 1). As expected, in absence of tubulin, Tau^MTSL^ is predominantly observed in the supernatant (Fig. 2A, lane 2). When we mixed Tau^MTSL^ with MTs, a band corresponding to Tau appeared in the pellet, indicating that labeling does not perturb its binding to MTs (Fig. 2A, lanes 3, 4, 5). Similar results were obtained when Tau was labeled with another spin label proxyl (Tau^proxyl^) (Fig. S1). Figure 2B shows turbidimetry time courses of MTs formation in the very specific experimental conditions determined in our previous work where Tau acts as an inducer of MTs formation at 37 °C^33^. In absence of Tau, 5 µM of tubulin (line 1) is not able to form MTs. When 5 µM of Tau is added to a same tubulin concentration in presence of the reducing agent TCEP used to avoid intra and/or inter covalent S-S bond (line 2), a drastic increase of absorbance at 350 nm is observed corresponding to the rapid formation of MTs. A similar result was obtained with Tau^MTSL^ (line 3) but with a lesser extent indicating that labeling slightly decreases the ability of Tau to induce MTs. Conversely, 5 µM of Tau^proxyl^ was not sufficient to induce MTs formation from 5 µM of tubulin (Fig. 2B, line 4). An increase of tubulin concentration to 20 µM was necessary to observe MTs formation (line 5). Note that alkylating Tau with iodoacetamide, a non-paramagnetic label that also binds covalently to cysteines but with a reduced steric hindrance compared to proxyl, reduce its efficiency to form MTs in classical conditions (5 µM tubulin), however with a lower efficiency than unlabeled Tau. Structures of formed MTs in each condition were checked by electronic microscopy (EM) and *bona fide* MTs were observed in correlation with turbidimetry signal (Fig. 2C). These results suggest that the presence of a non-cleavable group bound on the two cysteines of Tau decreases its capacity to induce MTs in a size label dependent manner, suggesting the presence of structural constraints in the Tau/tubulin binding site(s). It is worth noticing that in EPR experiments conditions, Tau and tubulin concentrations are higher (10 µM and 20 µM respectively) ensuring that MTs are present. As a consequence, Tau^proxyl^ and Tau^MTSL^ can be used to study local conformation modification or structural transition.

### Structural dynamics of Tau in interaction with MTs

**Figure 3:**
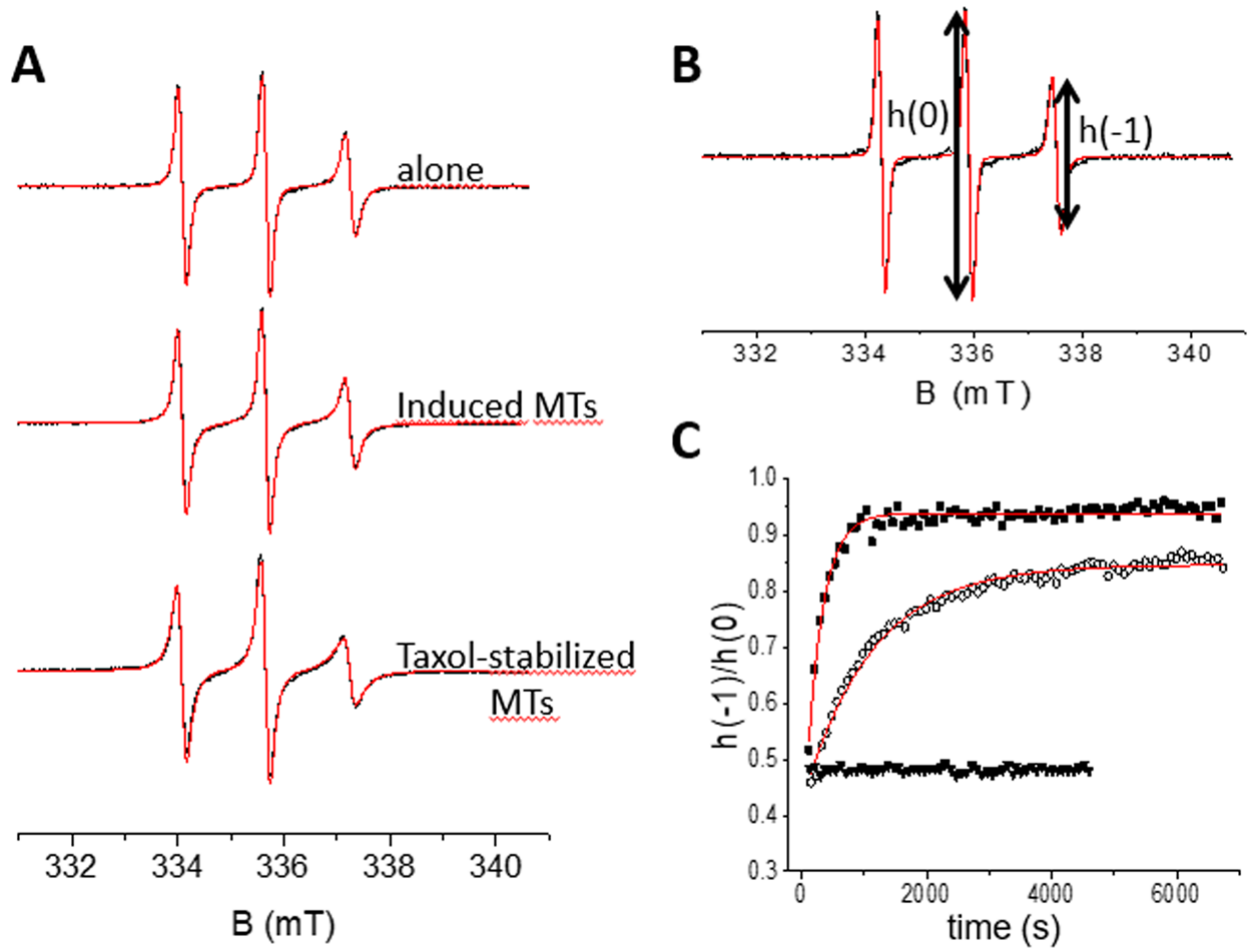
EPR spectra of labeled Tau alone and in presence of Microtubules. (**A**) EPR spectra (black) of Tau^proxyl^ alone, in presence of 20 µM tubulin and in presence of 20 µM Taxol-stabilized MTs superimposed with the simulated spectra (red). (**B**) EPR spectrum of Tau^MTSL^ alone (black) superimposed with the simulated spectrum (red). (**C**) h(−1)/h(0) ratio as a function of time of a 1:2 molar ratio of Tau^MTSL^:tubulin (■), of a 1:2 molar ratio Tau^MTSL^: Taxol-stabilized MTs (**o**) and of Tau^MTSL^ alone (▼). Data were fitted using the single-exponential curve y = y_o_ + (A − y_0_)*(1 − exp(−kt)) (red curves). Tau^MTSL^ concentration was 10 µM. Buffer: NaPi 20 mM pH 6.5, GTP 0.1 mM. Temperature: 37 °C.

EPR spectra of Tau^proxyl^ and Tau^MTSL^ are shown in Fig. 3A,B with the indication of the peak-to-peak amplitude of the central line h(0) and the high-field one h(−1). Composed of three narrow lines, these spectra are typical of a disordered protein where both the mobility of the label and the high flexibility of the side-chain contribute to the narrowing of the lines. The spectra can be simulated using a single component with rotational correlation times τ_c_ of 0.24 ± 0.02 ns and 0.19 ± 0.01 ns (Table 1) for Tau^proxyl^ and Tau^MTSL^ respectively, showing that the two labeled cysteine (291 and 322) environments are not discernible whatever the spin label. The difference in the value probably comes from a slight difference of mobility of the two labels. When the label was linked to Tau cysteine residues by a non-cleavable thioether bond (Tau^proxyl^), in presence of either tubulin or Taxol-stabilized MTs, we observed slight modifications of the EPR spectral shapes (Fig. 3A). For Tau^proxyl^-induced MTs, two components were required for the simulation, with τ_c_ values of 0.24 ± 0.02 and 0.76 ± 0.02 ns accounting for 19 and 81% respectively (Table 1). The first component corresponds to Tau^proxyl^ alone in solution, *i*.*e*. not bound to tubulin. The second component has a τ_c_ value which is slightly larger than the first component, but is still in the rapid regime of mobility. In presence of Taxol-stabilized MTs, the EPR spectrum can also be simulated using two components with τ_c_ = 0.24 ± 0.02 ns and 1.19 ± 0.02 ns accounting for 55 and 45% respectively. Again, the first component corresponds to a proportion of unbound Tau^proxyl^. Note that this proportion is in agreement with cosedimentation assay (Fig. 2A) where free Tau (supernatant) represented approximately the same amount as the MT-bound Tau (pellet). These τ_c_ values around 1 ns in the bound forms reveal that even in protein assembly, the labels were in the rapid regime of mobility. This demonstrates that Tau bound to MTs remains highly dynamics and disordered in the regions of its natural cysteine residues (repeats R2 and R3). Indeed a more drastic spectral change would have been expected if spin labels were directly involved in tertiary contacts or in the case of an induced folding^31,34–36^. These results are in agreement with a highly dynamic nature of Tau MTB domain in interaction with MTs as already described^13,20,27,28^, making Tau:MTs a typical example of a so-called “fuzzy complex” in which the IDP keeps in the bound state a high flexibility^37^. It is worth noticing that a more restricted environment of the labels (higher τ_c_ value) for Tau bound to Taxol-stabilized MTs was found compared to the Tau-induced MTs (Table 1). This result indicates that grafted labels experience a different local environment in the interaction between preformed and tubulin/induced-MTs.

**Table 1:** EPR simulation parameters: rotational correlation time τ_c_ (ns) and relative proportion of each spectral component (%). Mean axial g-tensor: g_x_ = 2.0083 ± 0.0003, g_y_ = 2.0061 ± 0.0005 and g_z_ = 2.0022 ± 0.0005.

Labeled protein	Component 1		Component 2	
	τ_C_ (ns)	%	τ_C_ (ns)	%
Tau^MTSL^	0.19 ± 0.01	100	—	—
Tau^proxyl^	0.24 ± 0.02	100	—	—
Tau^proxyl^ induced MTs	0.24 ± 0.02	19	0.76 ± 0.02	81
Tau^proxyl^ + Taxol-MTs	0.24 ± 0.02	55	1.19 ± 0.02	45
F4^MTSL^	0.13 ± 0.02	100	—	—
C322^MTSL^	0.16 ± 0.02	100	—	—
C291^MTSL^	0.07 ± 0.02	100	—	—

Mean axial hyperfine tensor: A_x,y_ = 0.43 ± 0.7 mT and A_z_ = 3.96 ± 0.1 mT.

### Evidence of a thiol disulfide exchange between Tau and tubulin

**Figure 4:**
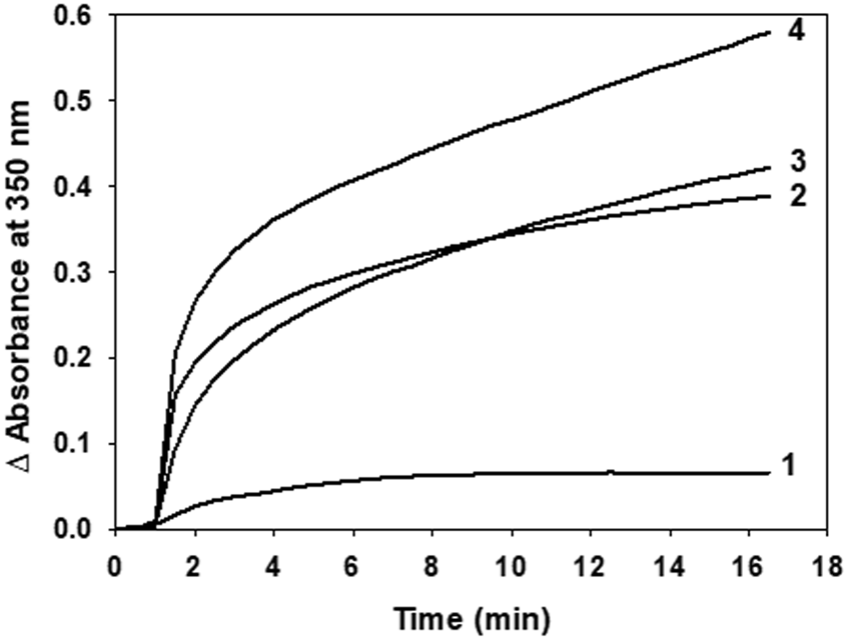
Turbidimetry analyses of Microtubule formation. Microtubule formation at 37 °C induced by (1) 2.5 µM of Tau^MTSL^, (2) F4^MTSL^, (3) C291^MTSL^, (4) C322^MTSL^ observed by turbidimetry. Tubulin concentration was 5 µM.

**Figure 5:**
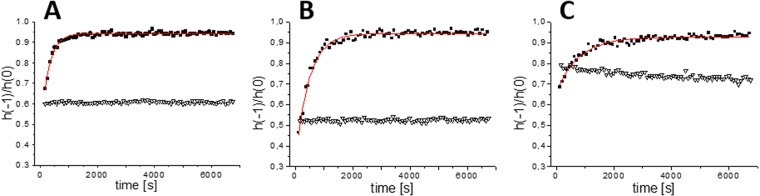
Kinetics of MTSL release for Tau fragments. h(−1)/h(0) ratio as a function of time of (**A**) F4^MTSL^, (**B**) F4 C291^MTSL^ and (**C**) F4 C322^MTSL^, alone (∇) and in presence of tubulin in a 1:2 molar ratio of F4:tubulin (■). Data were fitted using the single-exponential curve y = y_o_ + (A − y_0_)*(1−exp(−kt)) (red curves). F4^MTSL^ concentration was 10 µM. Buffer: NaPi 20 mM pH 6.5, GTP 0.1 mM. Temperature: 37 °C.

When Tau cysteine residues was linked to the label by a disulfide bond (Tau^MTSL^) and mixed to tubulin in a 1:2 molar ratio at 37 °C in order to form MTs, the EPR spectrum of Tau^MTSL^ unexpectedly evolves with time until reaching the spectral shape of free MTSL after approximately 25 min (Fig. 3C). Label release kinetics was measured by plotting the ratio h(−1)/h(0) as a function of time and data fitting with an exponential curve gave a k value of 0.23 min^−1^ (see Material and methods and Table 2). The same experiment has been performed by mixing Tau^MTSL^ with Taxol-stabilized MTs at 37 °C. As previously, the release of the label was observed, but with a slower kinetics: k = 0.054 min^−1^ (Fig. 3C and Table 2). As a control, the EPR signal of Tau^MTSL^ alone in solution has been measured in the same conditions and was shown to remain stable over time (Fig. 3C). To be sure that the release did not come from possible remaining of unlabeled Tau, the same experiment has been performed by mixing Tau^MTSL^ with Tau in a 1:2 molar ratio. In this case, no release of the label was observed (Fig. S2). To understand the role of the two natural cysteines of Tau in the label release observed upon interaction with tubulin, a truncated Tau fragment namely F4, and its two cysteine mutants were used^38^. These short constructs contain either the two cysteine residues at positions 291 and 322 (referred to as F4) or a single cysteine at position 322 (C291S mutant, referred to as C322^MTSL^ once labeled) or a single cysteine at position 291 (C322S mutant, referred to as C291^MTSL^ once labeled) (Fig. 1A). All these fragments were able to induce the formation of MTs (Fig. 4). Compared to Tau^MTSL^ (line 1), turbidimetry results show that all labeled F4 fragments are more active. Nevertheless, whereas C291^MTSL^ activity is similar to the F4^MTSL^ one (lines 2, 3), C322^MTSL^ has a higher activity (line 4). EPR spectra of F4^MTSL^, C322^MTSL^ and C291^MTSL^ can be simulated using a single component with rotational correlation time τ_c_ of 0.13 ± 0.02 ns, 0.16 ± 0.02 ns and 0.07 ± 0.02 ns respectively (Table 1 and Fig. S3). For F4^MTSL^, in which the labels are grafted at the same positions as Tau^MTSL^, the τ_c_ value is very close to the one obtained for Tau^MTSL^, showing that the truncated parts do not influence strongly the dynamics of the region containing the labels. Comparison between C322^MTSL^ and C291^MTSL^ shows that the two cysteine positions are not equivalent: label at position 291 experiences a more rapid mobility meaning that its environment is more flexible than at position 322. Note that the τ_c_ value for F4^MTSL^ is in between the two τ_c_ values obtained for the individual labeled cysteine residues. Figure 5 shows the time evolution of h(−1)/h(0) ratio for F4^MTSL^, C322^MTSL^ and C291^MTSL^ in presence of Tau-induced MTs. For each variant, label release is observed as in the case of the full Tau^MTSL^. Nevertheless, label release kinetics is different for the three F4 fragments. For F4^MTSL^, the k value (0.217 min^−1^) is similar to the full Tau^MTSL^ one whereas for C322^MTSL^ and C291^MTSL^, release kinetics are slower as indicated by their k values of 0.077 and 0.127 min^−1^ respectively (Table 2). As a control, stability over time of each sample alone in solution was checked (Fig. 5). Finally, we followed the label release for F4^MTSL^ in presence of Taxol-stabilized MTs (Fig. S4). Clearly, we observed a decrease in label release kinetics for F4^MTSL^ since the k value drops to 0.025 min^−1^ (Table 2). Label release occurs then almost 10 times slower when MTs are Taxol-stabilized.

**Table 2:** Fitting parameters of label release kinetics.

Labeled protein	Interaction conditions	y_0_	A	k (min^−1^)
Tau^MTSL^	Induced-MTs	0.29	0.94	0.23 ± 0.01
	Taxol-MTs	0.42	0.85	0.054 ± 0.002
F4^MTSL^	Induced-MTs	0.46	0.94	0.217 ± 0.009
	Taxol-MTs	0.44	0.84	0.025 ± 0.001
C291^MTSL^	Tau-MTs	0.32	0.95	0.127 ± 0.004
C322^MTSL^	Tau-MTs	0.66	0.93	0.077 ± 0.004

Data fitting has been done using the single-exponential curve y = y_o_ + (A − y_0_)*(1 − exp(−kt)), where y_o_ is the h(−1)/h(0) value at t = 0 min, A is the maximum value of h(−1)/h(0) and k is the kinetic constant (in min^−1^) of the label release. [Tau] = 10 µM, Tau/tubulin ratio of 1:2; Temp = 37 °C.

The release involves the breakage of the disulfide bond between the sulfur atoms of the cysteine Tau (or Tau fragments) and the label that implies the involvement of cysteines in the partner protein (tubulin). This has already been observed in the case of the chlorosplastic IDP CP12 in complex with GAPDH from *C*. *reinhardtii*^39^. MTSL release is the result of an oxydo-reduction mechanism that forms, at least in a first step, a covalent complex between Tau and tubulin *via* a disulfide bond. However, western blot analysis of Tau^MTSL^ and F4^MTSL^ in non-reducing condition did not show the presence of such Tau:tubulin complex (Fig. S5). This suggests that the covalent complex between Tau and tubulin is only transient and that the end of the reaction results in the formation of intra-molecular disulfide bonds within the tubulin. Tubulin disulfides have for a long time been reported to play a role in tubulin folding and thiol-disulfide exchanges proposed to be key regulators in MTs assembly and dynamics^40^. Wang *et al*. showed the presence of a disulfide bond between the α-C347 of α-tubulin and the cysteine of a synthetic stathmin-like peptide (Ncap) that prevents MTs formation^41^ and more recently, a thiol-disulfide exchange reaction between tubulin and GAPDH has been demonstrated^42^. Our result reveals for the first time the ability of Tau to perform a thiol/disulfide exchange with tubulin/MTs.

The functional activity of F4^MTSL^ to induce MTs was found to be much higher compared to Tau^MTSL^ (Fig. 4) whereas MTSL release kinetics were very similar (Fig. 5). This indicates that the two truncated parts (N-terminal projection domain and C-terminal part) play a role in Tau induced MTs formation and act as down-regulating regions. MTSL release kinetics measurements on F4 and its mutants show that C291 is more reactive compared to C322 (Fig. 5B,C). Conversely, functional studies point out that C322 is the most active to induce MTs, showing that thiol-disulfide exchange kinetics and activity are not correlated. Different aspects of the two natural cysteines of Tau have already been described in the literature. They have been found to form intra-molecular disulfide bridges preventing Tau to form the pathological Paired Helical Filaments (PHFs)^43^. They can also form inter-molecular bridges leading to a dimer acting as a seed for the initiation of Tau self-assembly in straight or PHFs *in vitro*^44,45^. Moreover it has been shown that cation binding such as Zinc on these two cysteines directly regulates Tau toxicity independently from phosphorylation^46^ and that they are also involved in an autoacetylation mechanism^47^. In the case of Tau binding on MTs, one of our previous study showed that formation of the intra-molecular disulfide bridges led to a partial detachment of the C-terminal part of Tau, and decreased significantly its overloading on the MTs surface^13^. Our study brings new insights into the role of the natural cysteine residues of Tau in its correct localization in the tubulin binding site necessary for its Microtubule inducer activity. The difference in the kinetics of label release related to the way MTs are formed and to the position of the Tau cysteines, in addition with the functional properties of the samples led us to search for the localization of Tau binding sites on tubulin.

### Tau Binding sites on tubulin/MTs

**Figure 6:**
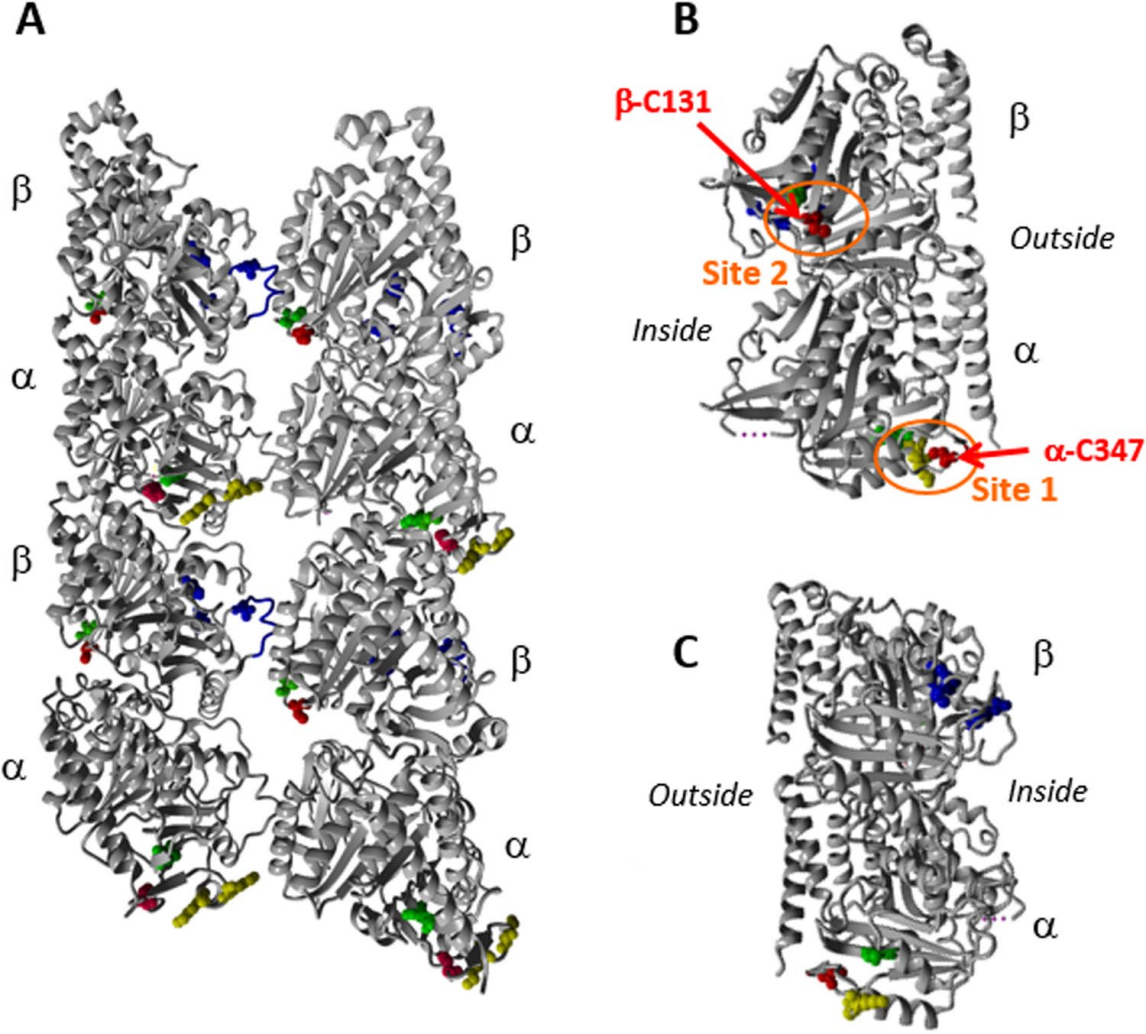
View of GDP-bound dynamic microtubules structure (**A**) Outside view of high-resolution cryo-EM structures (5.6 Å) of GDP-bound dynamic microtubules (pdb 3j6f). (**B**) Right view of only one Tubulin dimer of the MT. The two putative binding sites 1 and 2 of Tau on tubulin are encircled in orange. Cys347 on α-tubulin and Cys131 on β-subunit are highlighted in red. Cys129 on β-subunit and Cys315 and 316 on α-tubulin are represented in green. The three amino acids implicated in the Taxol binding pocket: His229, Thr276 and Arg369 and the M loop are represented in blue. Lys336 and 338 that are demonstrated to be crosslinked with Lys311 of Tau in Kavadath *et al*. are represented in yellow^27^. (**C**) Left view of only one Tubulin dimer of the MT. The figure is made with YASARA (Elmar Krieger, Gert Vriend; YASARA View—molecular graphics for all devices—from smartphones to workstations)^62^.

Thiol/disulfide exchange reactions between spin-labeled Tau and tubulin result necessarily in the formation of disulfide bridges within the tubulin since the presence of the Tau-SS-tubulin complex was not observed by western-blotting in non-reducing conditions (Fig. S5). Tubulin possesses 20 free cysteines distributed across both subunits (12 in α-tubulin and 8 in β-tubulin)^16,48^. The fact that the kinetics of label release was much slower in the case of preformed MTs compared to induced-MTs led us to carefully analyze the differences between the 3D reconstructions of MTs with (PDB code 3j6g) and without Taxol (PDB code 3j6f). Using PDBsum, we explored both longitudinal (α-β-α-β-…) and transversal interactions (α-α or β-β) in terms of residues involved in weak interactions^49^. The analysis of the longitudinal interactions of the Taxol-MTs structure showed the implications of numerous residues: 34 residues in α-subunit and 33 in the β-subunit involved in intra-dimer longitudinal interactions and 20 residues of α-subunit interact with 23 in the β-subunit in inter-dimer longitudinal interactions (Fig. S6). Among these residues we found the involvement of two cysteines: α-C347 with β-V181 and β-C131 with α-E97. On the other hand, only few residues were found in transversal interactions (data not shown) and among them no cysteine. Similar analyses were conducted on the structure of MTs without Taxol. In longitudinal interactions, the number of residues involved in the intra-dimer surface was slightly reduced (29 residues in α-subunit interact with 32 residues in the β-subunit) and the number of residues involved in the inter-dimer surface was increased (31 residues of α-subunit interact with 32 residues of the β-subunit of the adjacent dimer). More interestingly, no cysteine was found neither in longitudinal nor in lateral protofilament interaction. As a consequence, α-C347 and β-C131, which are less accessible in Taxol stabilized MTs, appear as good candidates for the location of Tau binding sites. In the MTs, α-C347 is localized at the inter-dimer interface whereas β-C131 is in the intra-dimer interface (Fig. 6). β-C131 is also accessible by passing through the MTs pore when the MTs are already formed (Fig. 6A). We then hypothesize, that the MTB domain, in particular the R2 and R3 repeats, bearing the Tau cysteines, are localized close to these two tubulin cysteines resulting in two binding sites (Fig. 6B).

As thiol/disulfide exchange results in the formation of disulfide bridges within tubulin, we examined the presence of other cysteines in the vicinity of α-C347 and β-C131. In α-subunit we found a couple of adjacent cysteines: α-C315 and α-C316 close to the α-C347 (Fig. 6) indicating the possibility to form a disulfide bridge in this region. This binding site (site 1) located at the interface between α-β heterodimers has already been proposed combining NMR and competition experiments with vinblastine^27^. Because there are two potential Tau binding sites on tubulin and also two natural cysteines on Tau, it is tempting to try to attribute the binding of one cysteine of Tau to one site of tubulin. As α-C347 has been shown to be the most reactive cysteine within the 20 cysteines in tubulin^50^, we propose that α-C347 interacts with Tau C291 because of its higher reactivity in thiol/disulfide exchange (Fig. 6). This hypothesis is reinforced by cross-linking experiments between Tau peptide containing only the C291 with α-K336 and α-K338 of the tubulin in the vicinity of α-C347 (Fig. 6)^27^. In β-subunit, β-C129 is very close to β-C131 involved in longitudinal contact in Taxol-stabilized MTs (Fig. 6), enabling the formation of C129-C131 disulfide bridge. By consequence we propose β-C131 as a second Tau binding site (site 2). This region of β-tubulin is close to the M-loop, an important secondary structure region for stabilization of the MTs, that protrudes from the protofilament inside the MTs^51^. By consequence, the region of Tau containing the C322 might bind the tubulin close to the M-loop which could explain the better activity in MTs formation of Tau fragments C322 compare to C291 (Fig. 4). β-C131 is also close to the Taxol binding site involving the three residues β-T276, β-H229 and β-R369 and located in the interior of the MTs^49^. Site 2 is also in agreement with competition experiments between Tau and Taxol suggesting a sharing of binding site on tubulin^19^. Moreover, the slower kinetics of label release for Tau fragment C322 is in agreement with the reduced activity of β-C131 and β-C129 reported in Britto *et al*. because of negative surroundings^50^. As a consequence, we propose to attribute Tau C322 as the cysteine involved in this second binding site.

In light of our results, we hypothesize that C291 acts as an anchor in the binding of Tau at site 1 of tubulin and thus favors the subsequent association of Tau at site 2. This is supported by the fact that in the different isoforms of Tau, Tau3R lacks the R2 repeat and thus the cysteine C291. It has been reported that this particular isoform is less efficient in its ability to interact with MTs and to stabilize them^52–55^. The alteration of the balance between Tau3R/Tau4R has been shown to be responsible for some neurodegenerative diseases^56^. Moreover, even in the Tau4R isoform, several mutations located in the R2 repeat have been shown to be involved in frontotemporal dementia and parkinsonism linked to chromosome 17. Among these mutations, the replacement of C291 into Arginine has been recently described^56^. In both cases, lack of Tau3R or C291R mutation, the absence of C291 leads to a defect in Tau/MTs association resulting in Tau aggregation.

### Conclusion

**Figure 7:**
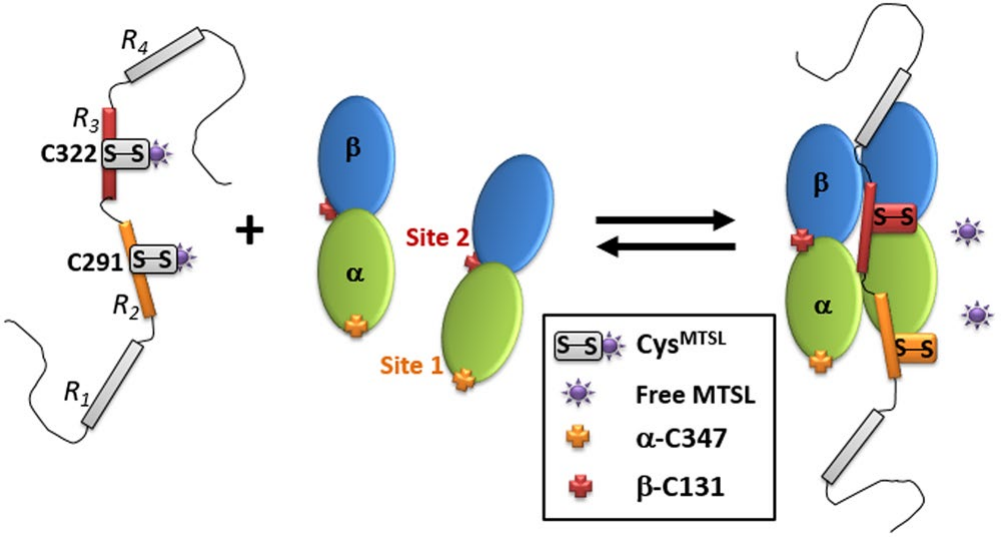
Model of Tau:tubulin interaction involving the Tau R2 repeat (C291) with the α-subunit of tubulin at Site 1 involving the region of α-C347 and the Tau R3 repeat (C322) with the β-subunit of tubulin at Site 2 involving β-C131 and β-C129.

The stabilization of MTs by Tau is a poorly understood mechanism and bringing insights into the molecular details characterizing this interaction is needed. The difficulty resides in the fact that on one side Tau is a long IDP (441 amino acids) and on the other Microtubules are complex assemblies. The unexpected release of the most commonly used MTSL spin labels from Tau induced by complex formation with tubulin/MTs led us to use SDSL-EPR in a very unconventional way. It is however worth noticing that even in this way, the technique can bring crucial information through the analyses of label release kinetics associated to structural data analyses. Our study and the recent literature allowed us to propose a Tau binding model involving two sites on tubulin associated to the two natural cysteines of Tau. We propose that Tau R2 repeat (more precisely C291) binds to the α-subunit of tubulin at site 1 involving the region of the most reactive cysteine known in tubulin α-C347 and that Tau R3 repeat (C322) binds to the β-subunit involving β-C131 and β-C129 (Fig. 7). Site 1 agrees with previous suggestions of binding site at the interface between tubulin heterodimer by competition experiments with vinblastine^27^. On the other hand, site 2 located close to the Taxol site inside the Microtubule has already been proposed^19^. Consequently, this model reconciles the previous models proposed so far.

## Methods

### Materials

Taxol was purchased from Alexis, and used without any purification. Sephadex G-25 medium was purchased from GE Healthcare (Uppsala, Sweden). All other chemicals were from Sigma Chemicals Co. (St Louis, MO, USA). PMSF and TCEP were from Sigma-Aldrich. Stock solution of PMSF was prepared in ethanol and stored at −20 °C. The spin labels 1-oxyl-2,2,5,5-tetramethyl-δ3-pyrroline-3-methyl methanethiosulfonate (MTSL) and 3-maleimido-2,2,5,5-tetramethyl-pyrrolidinyloxy (proxyl) were purchased from Toronto Research Chemicals Inc. and Sigma-Aldrich respectively.

### Tau purification

The 441 amino acids isoform of Tau, hTau40 (termed Tau throughout), was expressed from a pET vector (kindly provided by Dr M. Goedert). *E*. *coli* BL21(DE3) cells, bearing the plasmid were incubated overnight in 50 mL of LB medium containing 100 µg.mL^−1^ ampicillin at 37 °C with vigorous shaking. After 50-fold dilution of the culture in the same medium supplemented with 20 mM glucose and growth to OD_600nm_ of 1, the expression of the protein was induced by the addition of 0.75 mM isopropyl 1-thio-β-D-galactopyranoside, and cells were further incubated for 2h30 at 37 °C. Cells were harvested by centrifugation at 5000 g for 10 min at 4 °C and suspended in 2-(N-Morpholino)ethanesulfonic acid 45 mM, Triton X 100 8 mM, DTT 1 mM. After two runs in the French press (4 tones), the lysate was boiled at 90 °C during 12 min and centrifuged at 30000 g during 30 min. The supernatant was then passed through an exchange ion chromatography column Hi Trap SP-HP (34 µm − 2.5 cm × 1.5 cm-Vo = 5 mL) equilibrated with buffer containing 45 mM MES, pH 6.5. NaCl gradient was used to purify the protein that was detached with 0.25 M of NaCl. Fractions containing Tau were then pooled, dialyzed overnight to eliminate salt and dry-lyophilized. Tau was weighted and suspended before use in the desired buffer. Tau concentration was measured with a Perkin Elmer Lambda 800 UV/vis spectrometer using an extinction coefficient of 7700 M^−1^ cm^−1^ at 280 nm. Tau F4 fragments (aa 408–324) preparation, and its single mutants (C322S and C291S), followed the protocol described previously^57,58^.

### Tubulin purification

Tubulin was purified from lamb brains by ammonium sulfate fractionation and ion-exchange chromatography and stored in liquid nitrogen as described^59^. Before use, tubulin was equilibrated in appropriate buffer by passing through a desalting column of G25 (25 × 0.1 cm) and its concentration was determined spectrophotometrically at 275 nm with an extinction coefficient of 109000 M^−1^.cm^−1^ in 6 M guanidine hydrochloride.

### Taxol-stabilized MTs preparation

Tubulin was prepared as described above in 20 mM NaPi, GTP 0.1 mM pH 6.5 and diluted at the appropriate concentration. 20 µM of tubulin was incubated at 37 °C and MTs formation was induced by adding 8 mM MgCl_2_ and 25 µM of Taxol.

### Cosedimentation assay and SDS-PAGE

Taxol-stabilized MTs with or without Tau were centrifuged as described in Sillen *et al*. during 20 min at 88000 g at 20 °C to pellet MTs^13^. A cushion of glycerol was used during the centrifugation step to eliminate non-specific binding^52^. Tau concentration represents Tau bound to MTs in the pellet and free Tau in the supernatant. Polyacrylamide gel electrophoresis in denaturing conditions (SDS-PAGE) was performed using 12% acrylamide in the separating gel and Amersham Pharmacia low-weight calibration kit (97, 66, 45, 30, 20.1, and 14.4 kDa) for standards. Gels were stained with Coomassie brilliant Blue.

### MTs formation induced by Tau

Tubulin was prepared in 20 mM NaPi, GTP 0.1 mM pH 6.5 and diluted at the appropriate concentration. Samples were incubated at 37 °C, a sub-stoichiometric amount of Tau was added to induce MTs formation as previously described in Devred *et al*.^33^.

### Spin labeling

The labeling procedure of Tau and Tau fragments was performed in two steps: cysteine reduction and spin labeling. For cysteine reduction, 100 nmoles of Tau was incubated with TCEP (20 mM final) for 30 min in ice. TCEP was removed by gel filtration using a desalting PD-10 column (GE Healthcare) with an elution buffer of 20 mM sodium phosphate pH 6.5. After elution, the fractions containing Tau were pooled. Spin label (MTSL or proxyl) (Fig. 1B) was immediately added to the sample at 10 molar excess using a concentrated stock solution in acetonitrile. The reaction was carried out during 1 h and in an ice bath. Excess of unbound spin label was removed using a desalting column (PD-10) with the same elution buffer as in the previous step. The fractions containing the labeled Tau (Tau^MTSL^ or Tau^proxyl^) were pooled and concentrated under a continuous flow of argon at room temperature to evaporate water. Spin-labeled Tau was stored at −80 °C. Labeling on the two natural cysteine residues was checked by mass spectroscopy (Fig. S7).

### EPR spectroscopy and data analyses

EPR spectra were recorded on an Elexsys 500 Bruker spectrometer equipped with a Super High Q sensitivity resonator operating at X-band (9.9 GHz). All spectra were recorded at 37 °C using a Bruker N_2_ temperature controller (Bruker ER4131VT). The microwave power was 10 mW, the magnetic field modulation amplitude was 0.1 mT and the frequency modulation was 100 kHz. Spin concentration was measured by double integration of EPR signals compared to a reference sample (TEMPO solution at 104 µM). Labeling yields obtained were in the range of 150–200% for Tau^MTSL^, Tau^proxyl^, F4^MTSL^ (containing two cysteines) and in the range of 70% for C322S^MTSL^ and C291S^MTSL^ (containing one cysteine). For interaction experiments, labeled Tau (Tau^MTSL^ or Tau^proxyl^) or MTSL-labeled F4 fragments (10 µM) (Fig. 1A) were mixed with either tubulin or Taxol-stabilized MTs (20 µM) at 37 °C. For a detailed analysis of the EPR spectral shapes, simulations have been performed using SimLabel, a MATLAB Graphical User Interface (GUI) that uses some functions of the EasySpin toolbox^60^ and dedicated to multicomponent simulations of EPR spectra from SDSL-EPR experiments^34^. EPR spectral simulation allows decomposing the spectrum into different components and extracting, for each of them, the relative proportion and a dynamic parameter, namely, the rotational correlation time τ_c_. For label release kinetics, the evolution of the EPR spectral shape as a function of time was done by measuring the ratio of the peak-to-peak amplitude of the high- and central-field lines, referred to as h(−1)/h(0). This semi-quantitative parameter is highly sensitive to spin label mobility in the fast regime of mobility that is typically encountered in the studies of labeled IDPs^39^. Data fitting of label release has been done using the single-exponential curve y = y_o_ + (A − y_0_)*(1 − exp(−kt)), where y_o_ is the h(−1)/h(0) value at t = 0 min, A is the maximum value of h(−1)/h(0) and k is the kinetic constant (in min^−1^) of the label release.

### Tau alkylation

Tau was diluted in 0.1 M ammonium bicarbonate, 50 mM TCEP at 44 µM. For one volume of reduced Tau, two volumes of alkylation solution (10 mg/mL of iodoacetamide (Sigma) in 0.1 M of ammonium bicarbonate) are added and the sample was incubated for 30 min in the dark at room temperature. Then, one volume of 0.1 M ammonium bicarbonate, 50 mM TCEP was added. After 5 min of incubation, four volumes of H_2_O milliQ were added. Akylated Tau was dry-lyophilized and resuspended in the desired buffer. The alkylation was verified by mass spectrometry.

### Structure comparison

We used PDBsum, a database that provides an overview of the contents of each 3D macromolecular structure deposited in the Protein Data Bank. For each structure, PDBsum database includes analysis and schematic diagrams of protein–protein interactions^61^. We compared the high-resolution cryo-EM structures (4.7–5.6 Å) of microtubules stabilized by Taxol (pdb code 3j6g) to GDP-bound dynamic microtubules (pdb code 3j6f)^49^.

## Electronic supplementary material

Supplementary Information
