# Supplementary material for: Two Tau binding sites on tubulin revealed by thiol-disulfide exchanges
Source: Sci Rep. 2018 Sep 14;8:13846. doi: 10.1038/s41598-018-32096-9 (PMC6138654; doi:10.1038/s41598-018-32096-9)
Supplement: Supplementary file 1 — Supplementary Information [file 41598_2018_32096_MOESM1_ESM.docx]

**Supporting information**

**Two Tau binding sites on tubulin revealed by thiol-disulfide exchanges**

Marlène Martinho^1^, Diane Allegro^2^, Isabelle Huvent^3^, Charlotte Chabaud^1,2^, Emilien Etienne^1^, Hervé Kovacic^2^, Bruno Guigliarelli^1^, Vincent Peyrot^2^, Isabelle Landrieu^3^, Valérie Belle*^1^, Pascale Barbier*^2^.

1. *Aix-Marseille Univ, CNRS, UMR 7281 BIP, Bioénergétique et Ingénierie des Protéines, Marseille, France*
2. *Aix-Marseille Univ, CNRS UMR 7051, INP, Institut de Neurophysiopathologie, Marseille, France*
3. *Lille Univ, UMR 8576, CNRS, UGSF, 59000 Lille, France*

***** corresponding authors

**Figure S1.** MALDI-ToF mass spectrometry analysis (positive ion mode) of non-labeled Tau (black curve) and Tau^MSTL^ (red curve). Focus is made in the range of 44 000–50 000 Da. The mass increment corresponds to the mass of the two incorporated labels (2 x 186 Da).


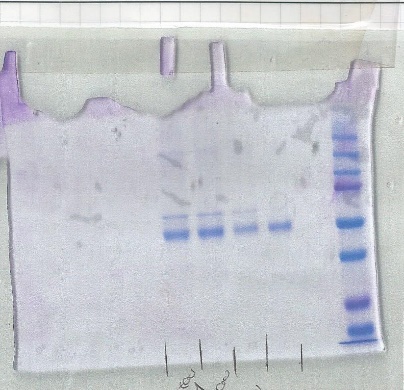

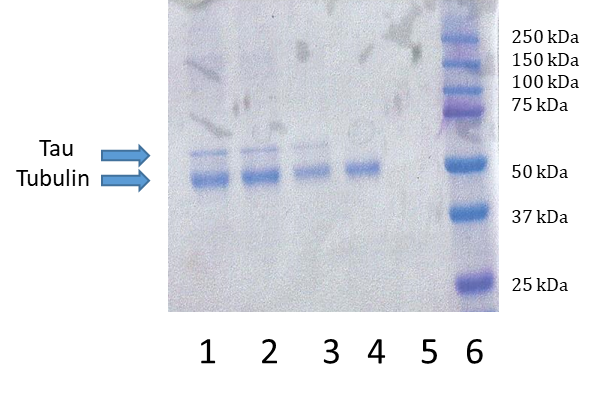


**Figure S2.** SDS-PAGE of pellet obtained after the centrifugation of 5 µM of Taxol- stabilized MTs with 5 µM (lane 1), 3.5 µM (lane 2), 2.5 µM (lane 3) of Tau^proxyl^. Taxol- stabilized MTs alone is in lane 4 and 5 µM of Tau^proxyl^ without tubulin in lane 5. The molecular mass markers are in lane 6.


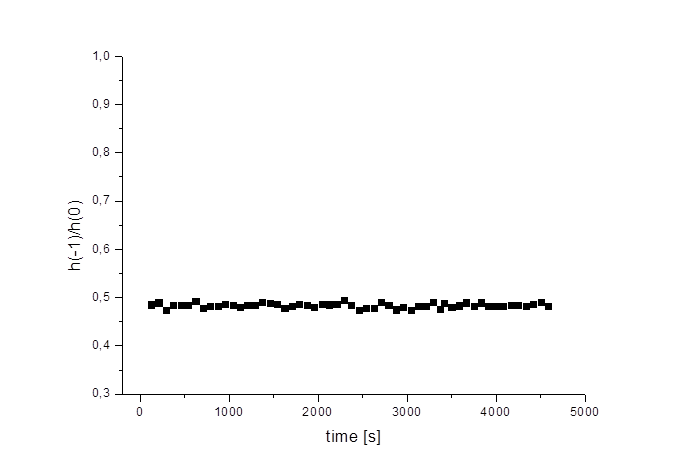


**Figure S3.** h(-1)/h(0) ratio as a function of time of a 1:2 molar ratio of Tau^MTSL^:Tau (⏹). Tau^MTSL^ concentration was 10µM. Buffer: NaPi 20 mM pH 6.5. Temperature: 37°C.


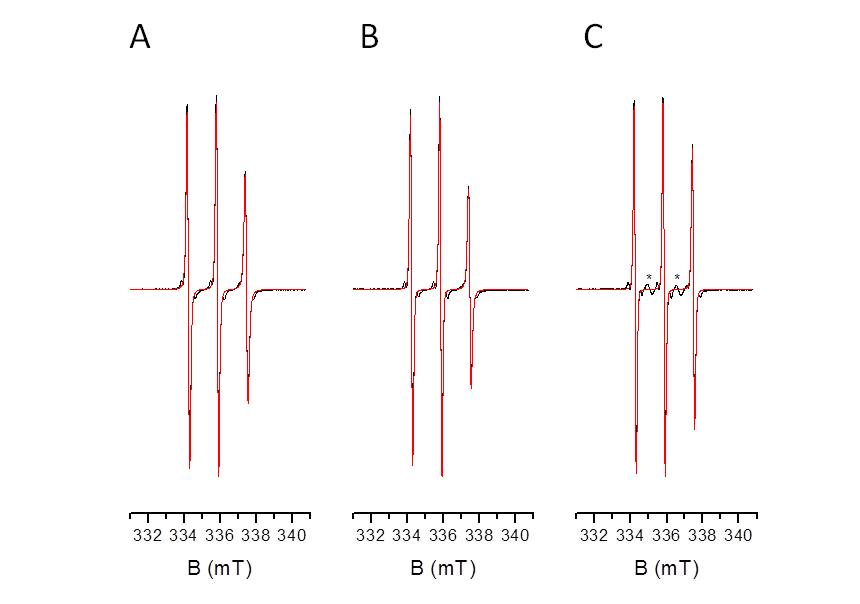


**Figure S4.** EPR spectra (black) of (A) F4^MTSL^, (B) C291^MTSL^ and (C) C322^MTSL^ superimposed with the simulated spectra (red) obtained using Easyspin toolbox^[1]^ and the Simlabel interface.^[2]^ Concentration was 10µM. Buffer: NaPi 20 mM pH 6.5. Temperature: 37°C. Star shows the presence of low amount (< 5%) of degradation of spin label in solution.


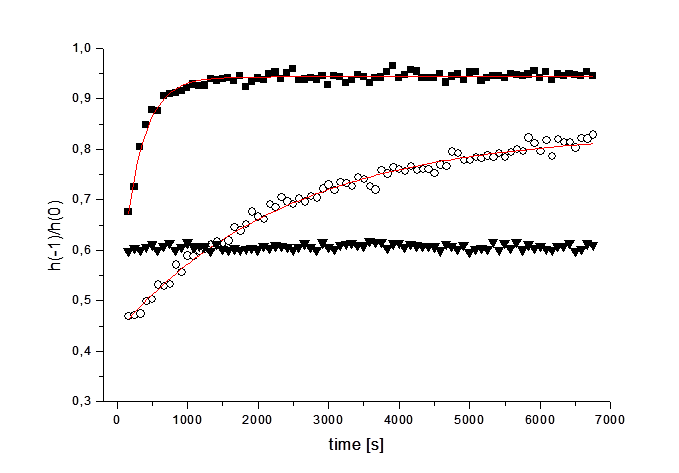


**Figure S5.** h(-1)/h(0) ratio as a function of time of a 1:2 molar ratio of F4^MTSL^:tubulin (⏹), of a 1:2 molar ratio F4^MTSL^: Taxol-stabilized MTs (o) and of F4^MTSL^ alone (⏷). Data were fitted using the single-exponential curve y = y_o_+(A-y_0_)*(1 - exp(-kt)) (red curves). F4^MTSL^ concentration was 10µM. Buffer: NaPi 20 mM pH 6.5. Temperature: 37°C.

Tubulin-Tau^MTSL^

Tubulin-Tau^MTSL^


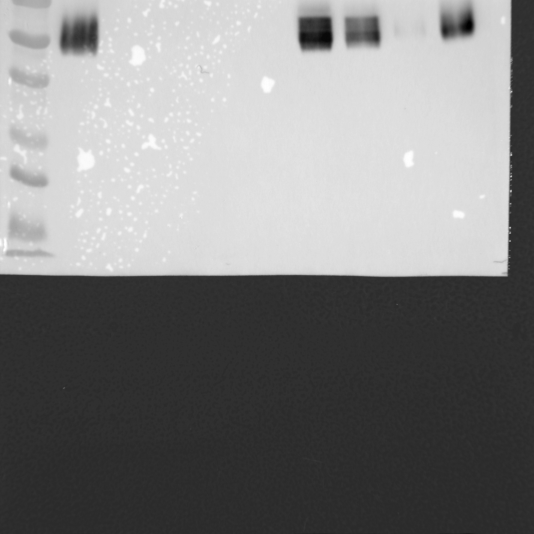

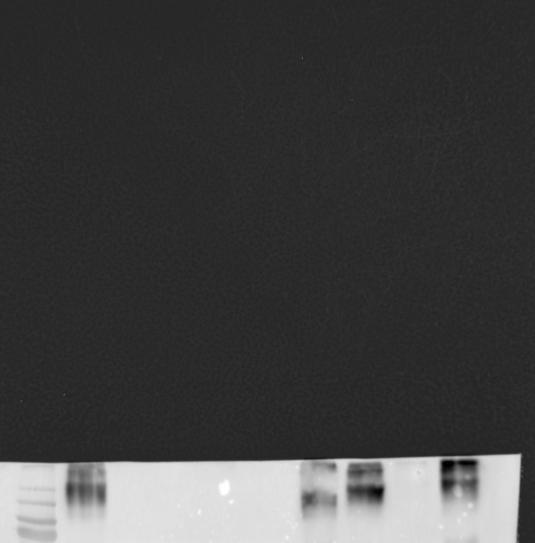

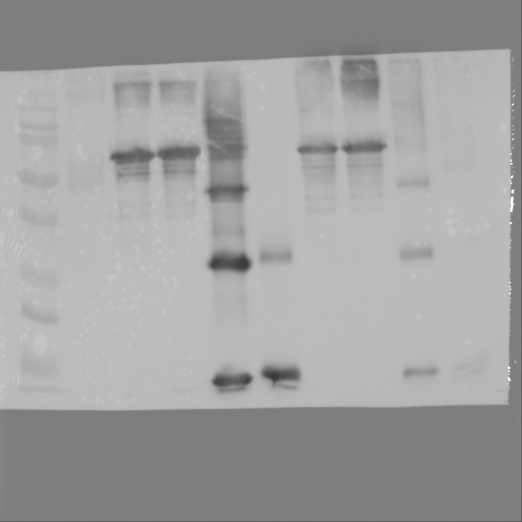


Anti-tubulin

Anti-tau

MW

MW

Tubulin

Tubulin

Tau

Tau

F4

Tau^MTSL^

Tau^MTSL^

F4

F4^MTSL^

F4^MTSL^

Tubulin-Tau

Tubulin-Tau

Tubulin-F4

Tubulin-F4

Tubulin-F4^MTSL^

Tubulin-F4^MTSL^

**Figure S6. A.** Anti-tubulin immunoblot (Antitubulin antibody T6199 was from Sigma and used at 1/1000) analysis of the supernatant obtained after centrifugation of microtubule with and without Tau and F4 Tau fragment (Tubulin, Tau and F4 Tau concentration were 5 µM). **B.** Anti Tau immunoblot (AntiTau antibody T1029 was from Euromedex and used at 1/500) of the same samples as in A. The revelation time is 1 min excepted for the part represented by dashed line. In this case the revelation time was 2 min. SDS-page is made in non-reducing condition to keep disulfide bond. The first lane is molecular mass markers. Dashed line shows the part of the blot that is cut and releveled with more revelation time.


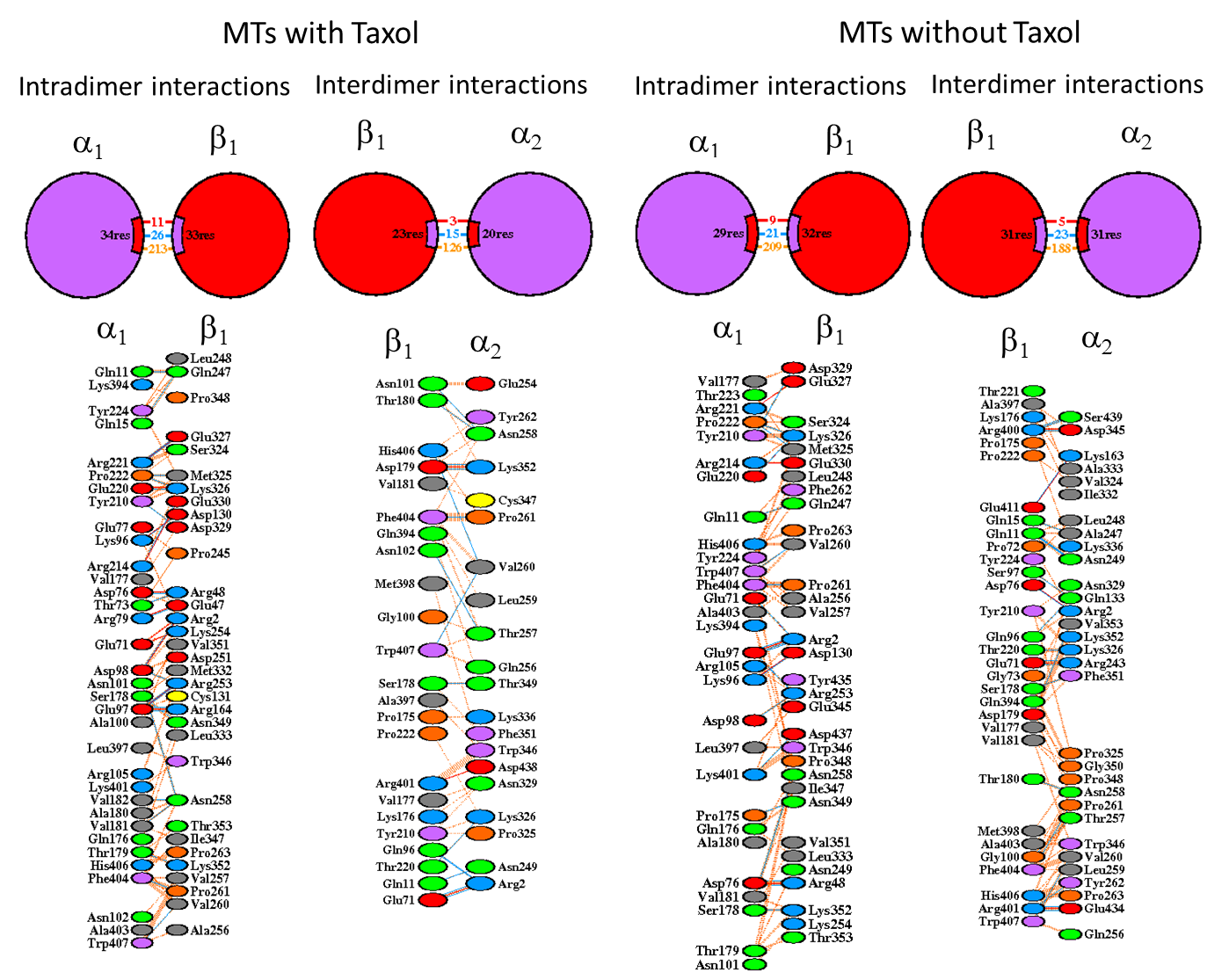


**Figure S7.** Comparison of longitudinal interaction between tubulin subunit with PDBsum in MTs in presence and absence of Taxol. Positive amino acids are represented in blue, negative in red, neutral in green, aliphatic in grey, aromatic in purple and cysteine in yellow. The number of H-bond lines between any two residues indicates the number of potential hydrogen bonds between them. For non-bonded contacts, which can be plentiful, the width of the striped line is proportional to the number of atomic contacts. The red line represents salt bridges, the yellow one disulfide bond and the blue line hydrogen bond. Orange lines represent non-bonded

Contacts.


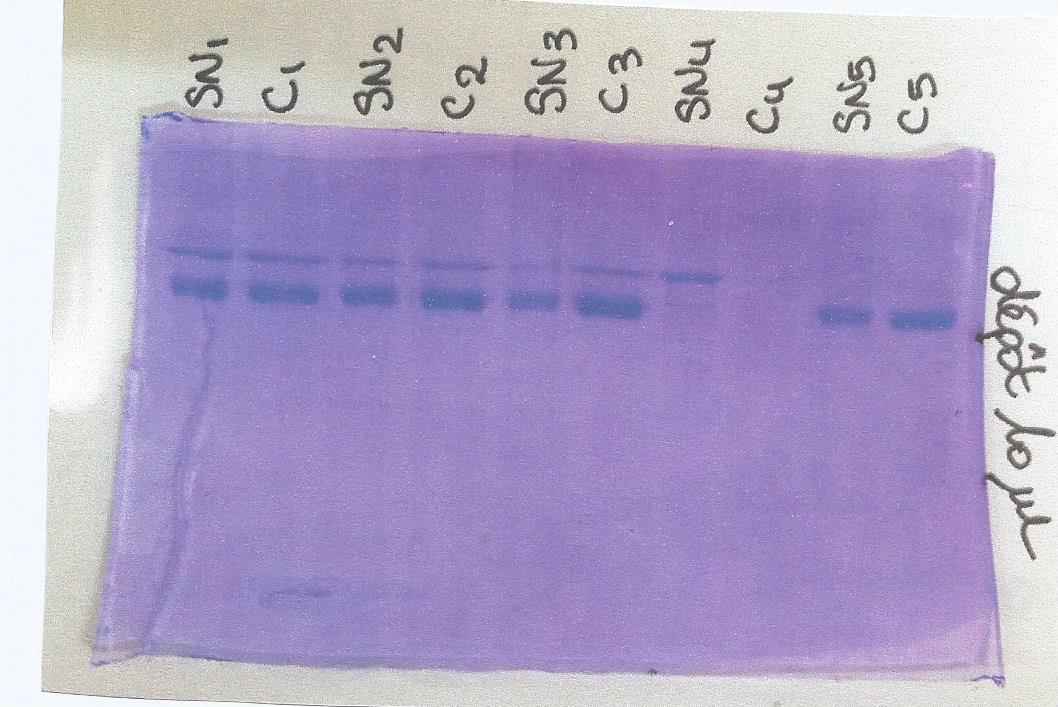


**Figure S8:** Original SDS-PAGE of figure 1A (dashed line). SN1 and C1 corresponds to the Supernatant and Pellet after centrifugation of 5 µM Taxol-stabilized Microtubules with 5 µM of Tau^MTSL^, respectively. SN2 and C2 corresponds to the Supernatant and Pellet after centrifugation of 5 µM Taxol-stabilized Microtubules with 3.5 µM of Tau^MTSL^, respectively. SN3 and C3 corresponds to the Supernatant and Pellet after centrifugation of 5 µM Taxol-stabilized Microtubules with 2.5 µM of Tau^MTSL^, respectively. SN4 and C4 corresponds to the Supernatant and Pellet after centrifugation of 5 µM of Tau^MTSL^, respectively. SN5 and C5 corresponds to the Supernatant and Pellet after centrifugation of 5 µM of 5 µM Taxol-stabilized Microtubules, respectively.

[1] S. Stoll, A. Schweiger, EasySpin, a comprehensive software package for spectral simulation and analysis in EPR, J Magn Reson, 178 (2006) 42-55.

[2] E. Etienne, N. Le Breton, M. Martinho, E. Mileo, V. Belle, SimLabel: a graphical user interface to simulate continuous wave EPR spectra from Site-Directed Spin Labeling experiments, Magn Reson Chem, (2017) doi: 10.1002/mrc.4578.
